# A glance at the prevalence of coronavirus disease 19 (COVID-19) in Iran: Strengths and weaknesses

**DOI:** 10.1017/ice.2020.193

**Published:** 2020-05-04

**Authors:** Nima Mohammadzadeh, Mahla Shahriary, Neda Shirmohammadlou, Vahid Lohrasbi

**Affiliations:** 1 Department of Microbiology, Faculty of Science, Shahid Beheshti University, Tehran, Iran; 2 Department of Microbiology, Faculty of Medicine, Tarbiat Modares University of Medical Sciences, Tehran, Iran; 3 Department of Microbiology, Faculty of Medicine, Zanjan University of Medical Sciences, Zanjan, Iran; 4 Student Research Committee, Iran University of Medical Sciences, Tehran, Iran; 5 Department of Microbiology, Faculty of Medicine, Iran University of Medical Sciences, Tehran, Iran

*To the Editor—*After epidemics of severe acute respiratory syndrome-related coronavirus (SARS) in 2002 and Middle East respiratory syndrome–related coronavirus (MERS) in 2012, the third global challenge from a coronavirus began at the end of 2019, but this time in the form of a pandemic.^1^ The coronavirus disease 2019 (COVID-19) is a respiratory tract infection caused by a virus closely related to the SARS virus called severe acute respiratory syndrome coronavirus 2 (SARS-CoV-2). According to the World Health Organization (WHO) guideline, patients with COVID-19 have symptoms ranging from mild respiratory illness (respiratory symptoms, fever, cough, shortness of breath, and breathing difficulties) to severe (pneumonia, acute respiratory distress syndrome, kidney failure, and death).^1^ Based on scientific reports,^2^ this pandemic began in Wuhan City, China, and quickly spread throughout the country. As of March 15, China has confirmed 81,048 cases with 3,204 deaths. Although the COVID-19 incidence has declined sharply since February 13, 2020, in China, the prevalence increased faster in other countries than scientists anticipated. Italy, with 21,157 positive cases and 1,441 deaths (case fatality rate [CFR], 6.81%), and South Korea with 8,162 positive cases and 75 death (CFR, 0.91%), were the countries most affected as of March 15.^2^

In Iran, the first COVID-19 case was identified on February 19, 2020, in Qom. As of March 16, 2020, 14,991 COVID-19 cases with 853 deaths (CFR, 5.69%) have been reported cumulatively from all 29 provinces of Iran. Among these states, Tehran has shown the highest incidence, with 3,774 positive cases.^2^ Although Iran has achieved more success in controlling and preventing patient deaths than some developed countries, such as Italy (CFR, 6.81%), Iran has faced some difficulties in controlling this epidemic. Thus, achieving success and overcoming COVID-19 in Iran requires that we understand these deficiencies.

Some of the most effective actions in Iran in the early days of the COVID-19 outbreak included the following: (1) encouraging people to stay home, (2) providing free diagnostic and therapeutic services to COVID-19 patients, (3) allocating at least 1 hospital in each province to providing special services to COVID-19 patients, (4) daily disinfecting public transportation like subways, buses, and taxis, (5) canceling sport competitions, cinemas, theaters, weddings, and funerals, and closing all schools and universities, (6) providing distance and online learning infrastructure for students (7) increasing the capacity of the production of masks and disinfectants by >7 times, (8) extensively informing and promoting a culture of healthcare by the Islamic Republic of Iran Broadcasting system and other media, and launching mobile software, websites and telephone answering systems to address concerns and questions about COVID-19, (9) encouraging people to eat homemade healthy foods instead of fast food, (10) mobilizing and recruiting all national government and nongovernmental organizations to fight COVID-19 under the auspices of “The National Headquarters for Coronavirus,” (11) governmental support of small and large businesses to compensate for the loss of revenue and jobs, and finally (12) publication of a COVID-19 surveillance guideline by the Ministry of Health for various organizations (eg, prisons, barracks, etc) to stop and reverse the growing epidemic trend (Table 1).^2,3^

**Table 1.** List of All Actions Taken in Iran by Date and Category

Date	Policy and/or Action
19 Feb	• Established contagious disease management and control teams in Qom province • Assigned a hospital in Qom for influenza-like illness • Equipped negative pressure isolation section for isolation of suspicious cases
20 Feb	• Increased the production capacity of the mask to 1.2 million units per day
21 Feb	• Established the “Corona Virus Headquarters and Prevention” at the Ministry of Health • Obtained the “COVID-19 Laboratory Special Kit” from the World Health Organization • Inspected Tehran and Qom pharmacies to prevent hoarding
22 Feb	• Increased the production capacity of the face mask by working around the clock • Prohibited mask exports until further notice • Began daily disinfection of public transportation and their stations • Entered educational hospitals affiliated with medical universities to accelerate service delivery
23 Feb	• Supreme leader of Iran and Shia and Sunni Islam authorities start to help Ministry of Health • Prohibited hookah supplies • Cancelled student group programs
24 Feb	• Formed “the National Headquarters for Coronavirus” by president of Iran • Changed all exam dates
25 Feb	• Enlisted students and professors of pharmacy to make disinfectant solution • Enlisted the Army to help the Ministry of Health • Closed all centers for the celebration of the bride and groom until secondary notice
26 Feb	• Received 5,000 COVID-19 detection kits and masks from China
27 Feb	• Increased imports of all kinds of masks, gloves, and medical clothes • Increased travel restrictions • Began distributing disinfecting gels at 700 pharmacies in Tehran
28 Feb	• Increased medical alcohol production >3 times • Discovered a warehouse with 6 million gloves in Tehran by the Ministry of Intelligence
29 Feb	• The *COVID-19 Surveillance Guideline* was released by the Ministry of Health • Set up an answering center to answer questions about COVID-19 • Allocated 2 billion rials (US$47,500) to the Ministry of Health
1 Mar	• Equipped the Pasteur Institute of Iran for daily 12,000 detection test of COVID-19
2 Mar	• Enlisted police to help Ministry of Health with full capacity • Controlled and reduced traffic congestion in cities using police
4 Mar	• Special regulations for filling stations, restaurants, and public places • Abolition of prayer in all provinces
5 Mar	• Launched patient self-assessment and registration system
6 Mar	• Began providing daily 4,000 isolation uniforms for the treatment staff
7 Mar	• Sent 300 million test messages (SMS) from the Ministry of Health for prevention and health care
8 Mar	• Temporarily closed of all places of worship and pilgrimage
9 Mar	• Started clinical trial of Iranian COVID-19 drugs • Began checking the body temperature of all people at the entrances of public and nongovernmental centers and at the entrances and exits of cities • Printed and distributed brochures of “Environmental Control Guide to Combat COVID-19” in mass population centers (prisons, garrisons, and military and law enforcement centers), bus stations, train stations, and airport stations • Printed and distributed brochures of “Mental Health Guide in Crisis” to manage personal stress
11 Mar	• Called all volunteer doctors and paramedics to dispatch them to areas in need
13 Mar	• Issued order to build special healthcare facilities for patients with COVID-19 • Issued immediate import instructions for medical equipment and supplies and bypassing US sanctions
14 Mar	• Launched new coronavirus detection laboratory at the Pasteur Institute of Iran • Issued new restrictions on intercity traffic • Launched telephone psychological services
15 Mar	• Began delivery of thousands of 250,000 L disinfectant to medical universities • Launched the largest production line of N95 respirators and surgical masks in Iran by Ministry of Defense and Armed Forces Logistics
16 Mar	• Established a nursing care center after discharge of patients with a 780-bed capacity in Qom province

Iran has established an acceptable track record in the control of infectious diseases; but it still has very big challenges to reach an ideal level. These challenges include the following^2,4^: (1) imposition of travel restrictions by the government on religious cities, like Mashhad and Qom, is difficult; (2) approaching ancient Nowruz ceremonies, like Christmas, are traditionally associated with shopping and large gatherings of people; (3) a failure to quarantine the first city with positive cases of COVID-19 (Qom) with subsequent spread of infection to most provinces of Iran was hampered by the Islamic Consultative Assembly election of 2020 and lack of cooperation by the people; (4) lack of attention by the people to government warnings about travel to Gilan, Mazandaran, and Golestan provinces (popular holiday destinations in Iran) after the closure of schools and universities to aid in spreading the infection; (5) lack of money, medical equipment, and laboratory diagnostic kits because of international sanctions; (6) hoarding of medical devices such as gloves, masks, and disinfectants by profiteers; and (7) lack of space for the quarantine of people with suspected infection in the early days of the outbreak.

Finally, the number of cases in Iran places it in the statistical middle of the COVID-19 outbreak countries (Fig. 1). If Iran tries to overcome to all challenges that are described above, it will be able to manage this crisis. We daresay that “where there’s a will, there’s a way,” and we hope that these challenges will be overcome soon.

**Fig. 1.**
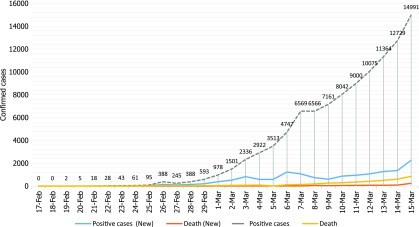
COVID-19 prevalence in Iran.

## Supplementary material

For supplementary material accompanying this paper visit http://dx.doi.org/10.1017/ice.2020.193. click here to view supplementary material
