# Supplementary material for: A glance at the prevalence of coronavirus disease 19 (COVID-19) in Iran: Strengths and weaknesses
Source: Infect Control Hosp Epidemiol. 2020 May 4:1–3. doi: 10.1017/ice.2020.193 (PMC7237388; doi:10.1017/ice.2020.193)
Supplement: Supplementary file 1 [file S0899823X20001932sup001.docx]

Table 1: List of all actions taken in Iran by date and category.

| Date | Policy and/or Action |
| --- | --- |
| 19 Feb | - Establishment of contagious disease management and control teams in Qom province - Assign a hospital in Qom for influenza-like illness - Equipping negative pressure isolation section for isolation of suspicious cases |
| 20 Feb | - Increasing the production capacity of the mask to 1.2 million units per day |
| 21 Feb | - Establishment of “Corona Virus Headquarters and Prevention” at the Ministry of Health - Get the “COVID-19 Laboratory Special Kit” from WHO - Inspecting Tehran and Qom pharmacies to prevent hoarding |
| 22 Feb | - Increasing the production capacity of the face mask by working around the clock - Prohibit mask exports until secondary notice - Daily disinfection of public transportation and their stations - Entering educational hospitals affiliated with medical universities to accelerate service delivery |
| 23 Feb | - Supreme leader of Iran and Shia and Sunni Islam authorities start to help Ministry of Health - Prohibition of hookah supply - Canceling student group programs |
| 24 Feb | - Formation of “the National Headquarters for Coronavirus” by president of Iran - Changing all exams dates |
| 25 Feb | - Movement of students and professors of pharmacy to make disinfectant solution - Army start to help Ministry of Health - Closing all centers for the celebration of the bride and groom until secondary notice |
| 26 Feb | - Receive 5,000 COVID-19 detection kit and mask from Chinese |
| 27 Feb | - Increasing import all kinds of masks, gloves and medical clothes - Increase travel restrictions - Distribution of disinfecting gels at 700 pharmacies in Tehran |
| 28 Feb | - Increasing medical alcohol production more than 3 times - Discover a warehouse with six million gloves in Tehran by the Ministry of Intelligence |
| 29 Feb | - The COVID-19 Surveillance Guideline was released by Ministry of Health - Setting up an answering center to people's questions about COVID-19 - Allocating 2 billion rials to the Ministry of Health |
| 1 Mar | - Equipping the Pasteur Institute of Iran for daily 12,000 detection test of COVID-19 |
| 2 Mar | - Police start to help Ministry of Health with full capacity - Controlling and reducing traffic congestion of cities by police |
| 4 Mar | - Special recipes for filling stations, restaurants and public places - Abolition of prayer in all provinces |
| 5 Mar | - Launch of patient self-assessment and registration system |
| 6 Mar | - Providing daily 4,000 isolation uniforms for the treatment staffs |
| 7 Mar | - Sending 300 million SMS by the Ministry of Health for the prevention and the healthcare |
| 8 Mar | - Temporary closure of all places of worship and pilgrimage |
| 9 Mar | - Start of clinical trial of Iranian COVID-19 drugs - Checking the body temperature of all people at the entrances of public and non-governmental centers and at the entrances and exits of cities - Printing and distributing brochures of “Environmental Control Guide to Combat COVID-19” in mass centers (prisons, garrisons, and military and law enforcement centers), bus stations, train stations, and airport stations - Printing and distributing brochures of “Mental Health Guide in Crisis” to managing people stress |
| 11 Mar | - Calling all volunteer doctors and paramedics to dispatch to needed areas |
| 13 Mar | - Order to build special health care facilities for patients with COVID-19 - Immediate import instructions for medical equipment and supplies and bypassing US cruel sanctions |
| 14 Mar | - Launch of new coronavirus detection lab at the Pasteur Institute of Iran - New restrictions on intercity traffic - Launching telephone psychological services |
| 15 Mar | - Delivery of thousands of 250,000 liters of disinfectant to medical universities - Launching the largest production line of N95 respirators and surgical masks in Iran by Ministry of Defence and Armed Forces Logistics |
| 1. Mar | - Establishment of nursing care center after discharge of patients in Qom province with 780 beds capacity |

|  |  |
| --- | --- |
